# Mitochondrial Genome (mtDNA) Mutations that Generate Reactive Oxygen Species

**DOI:** 10.3390/antiox8090392

**Published:** 2019-09-11

**Authors:** Anne Hahn, Steven Zuryn

**Affiliations:** Clem Jones Centre for Ageing Dementia Research, Queensland Brain Institute, The University of Queensland, 4072 Brisbane, Australia

**Keywords:** mitochondrial DNA (mtDNA), oxidative stress, reactive oxygen species, mitochondrial disease, aging, cancer, neurodegeneration

## Abstract

Mitochondria are critical for the energetic demands of virtually every cellular process within nucleated eukaryotic cells. They harbour multiple copies of their own genome (mtDNA), as well as the protein-synthesing systems required for the translation of vital subunits of the oxidative phosphorylation machinery used to generate adenosine triphosphate (ATP). Molecular lesions to the mtDNA cause severe metabolic diseases and have been proposed to contribute to the progressive nature of common age-related diseases such as cancer, cardiomyopathy, diabetes, and neurodegenerative disorders. As a consequence of playing a central role in cellular energy metabolism, mitochondria produce reactive oxygen species (ROS) as a by-product of respiration. Here we review the evidence that mutations in the mtDNA exacerbate ROS production, contributing to disease.

## 1. The Mitochondrial Genome

**Figure 1:**
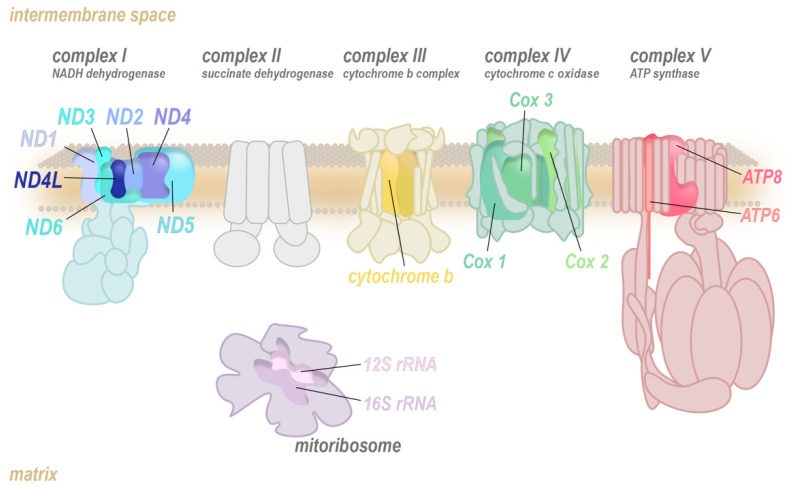
Subunit composition of each mitochondrial respiratory complex.

Mitochondria are a remarkable hub of metabolic activity, charged with producing the majority of the cell’s primary energy substrate, adenosine triphosphate (ATP). Most ATP is generated via oxidative phosphorylation (OXPHOS), a process involving the transfer of electrons from reduced nicotine adenine dinucleotide (NADH) or flavin adenine dinucleotide (FADH_2_) to O_2_ through a series of highly conserved multiprotein ensembles known as the electron transport chain (ETC; complexes I to IV; Figure 1). The energy released from these redox reactions is stored as a pH gradient and an electrical potential across the mitochondrial inner membrane, which is tapped by an enzymatic rotary mechanical motor, ATP synthase (complex V), to drive phosphorylation of adenosine diphosphate (ADP) into ATP.

The OXPHOS complexes are heteromultimers that are assembled from nuclear-encoded (muted colour) and mitochondrially encoded core subunits (bright colour). The translation of the mitochondrially encoded transcripts of the ETC is completed by the mitoribosome (purple) which also consists of mitochondrially and nuclear-encoded components.

**Figure 2:**
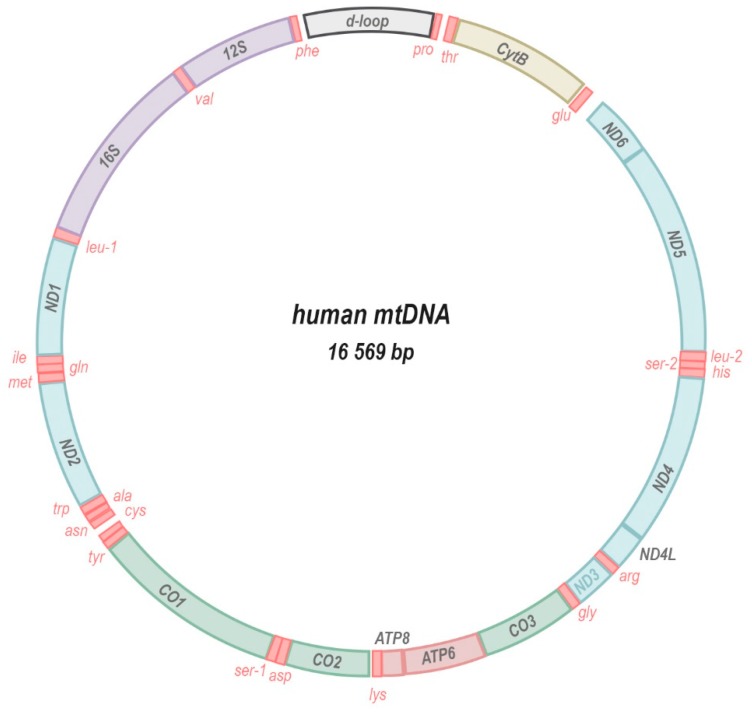
The mitochondrial DNA (mtDNA).

In humans, thirteen core subunits of the ETC (complexes I, III, and IV) and ATP synthase (F_1_F_0_-ATPase) are encoded by the mitochondria’s own genome (mitochondrial DNA, mtDNA; Figure 2). In addition, the mtDNA encodes ribonucleic acid (RNA) machinery (22 transfer RNAs, tRNAs; and 2 ribosomal RNAs, rRNAs) that is required for the translation of each subunit within the mitochondrial compartment. A remnant of their endosymbiotic origin, the mitochondrial genome is a small circular double-stranded DNA molecule ranging in size from 10 to 20 kilobases in the animal kingdom [1]. In plants, mtDNA is often longer, up to ~1700 kilobases, non-circular, and in some species is divided into several separate chromosomes [2]. Usually, multiple copies of the genome are present in each individual mitochondrion and hundreds to thousands of mtDNA copies may populate a network of fusing and budding organelles within a single cell. The presence of multiple copies of the mtDNA per cell (polyploidy) allows for different alleles or mutations—that are either inherited or arise sporadically—to be present in only a fraction of the total pool of mitochondrial genomes in a given cell or organism. This state, termed heteroplasmy, implies that the presence of a certain mtDNA mutation could be tolerated and inconsequential if it occurs at low enough fractions of the total pool. At higher heteroplasmy levels that exceed a certain biochemical threshold [3], the same mutation may be extremely deleterious or even lethal to an individual. This threshold is dependent upon many criteria such as the nature of the mutation, as well as the cell and tissue type. Moreover, through a combination of deterministic and stochastic mechanisms [4], heteroplasmy levels may differ significantly between tissues and cells of the same individual, resulting in complex phenotypic manifestations [5,6,7,8,9].

The mtDNA molecule encodes 13 crucial protein subunits of the electron transport chain (blue, yellow, green) and ATP synthase (muted red), 2 rRNA components of the mitoribosome (purple) and 22 mitochondrial tRNAs (bright red). Very little intergenic space separates the genes that are transcribed as a polycistronic transcript and also contain no introns. The regulatory region, called D-loop, comprises sites for the initiation of replication and transcription of the mtDNA.

Compared to the nuclear genome, the structure and composition of the mitochondrial genome is unique in several ways. Genes encoded by the mtDNA lack introns and utilise different nucleotide codons for certain amino acids (AUA encodes methionine instead of isoleucine [10] and UGA encodes tryptophan instead of a stop codon [10,11]), the stop codon (AGA and AGG encodes a stop codon instead of arginine), and the start codon (AUU encodes a methionine start codon instead of isoleucine [12]. Codon usage may also differ between species [13,14], such as in platyhelminthes where AGA and AGG encode serine instead of arginine [15]. Generally, the mitochondrial genome is controlled by a single regulatory sequence. This non-coding A-T-rich region of the mtDNA harbours two transcription start sites, one each on the light and heavy DNA strands, where the synthesis of polycistronic transcripts is initiated [16,17,18]. The same region also acts as an origin of replication for the mitochondrial genome. Interestingly, mtDNA replication often aborts soon after initiation, leaving the template strand, daughter strand and displaced lagging strand, a structure referred to as displacement (or D-) loop [19].

The transfer of genes to the nuclear genome and the minimal use of inter- and intra-genic material may have evolved to minimise the overall probability of the mtDNA acquiring nucleotide damage in the highly oxidative microenvironment of the mitochondria. However, there is wide consensus that mtDNA mutates much faster than nuclear DNA, although predictions of the mtDNA mutation rate have varied by four orders of magnitude [20]. Direct estimates in *Drosophila* using mutational accumulation lines suggest that mtDNA has a mutation rate 10–70-fold greater than that of the nuclear DNA [21]. The prevailing view has been that this higher mutation rate is attributable to damage caused by reactive oxygen species (ROS) formed by the leakage of electrons from the ETC onto molecular oxygen. The mitochondrial genome’s close spatial proximity to the sites of ETC-mediated ROS production make it particularly vulnerable to their damage. Moreover, mtDNA is not protected by histones that can organize DNA into highly compacted structures that offer protection. Despite this, it has become increasingly apparent that not ROS-mediated damage, but rather copying errors introduced into the mitochondrial genome during replication represent the major contributor to the high mtDNA mutation rate [22].

Replication of the mitochondrial genome is accomplished by the mitochondrion-specific polymerase γ, and, unlike the nuclear genome, is independent of cell division. MtDNA replication occurs at a frequency much greater than that of the nuclear genome and continues in postmitotic cells, increasing the likelihood of introducing copying errors in the sequence. Although polymerase γ has a proofreading activity, many of the other DNA repair mechanisms that maintain the integrity of the nuclear genome seem to be absent or less efficient within mitochondria [23]. Regardless of how mtDNA mutations are formed, a consequence of the compact genetic configuration of the mtDNA is the increased likelihood that any mutation will impinge upon a coding sequence and may thus alter the functional integrity of a vital protein or RNA. As such, potentially pathogenic mutations are relatively common in humans with an estimated prevalence of 1:200 in Western populations [24], albeit at low heteroplasmy levels.

In the following sections, we will discuss specific examples of mtDNA mutations that have been experimentally demonstrated to enhance ROS production and their links to human disease.

## 2. Mitochondrial-Encoded Complex I (NADH Dehydrogenase) Mutations

**Figure 3:**
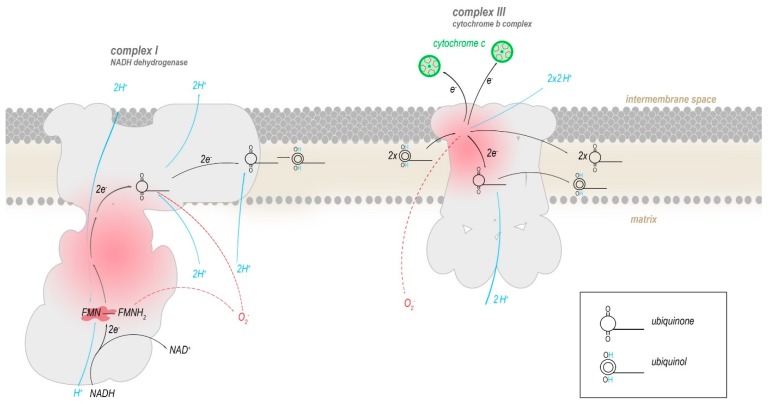
Complex I and III interaction with ubiquinol.

Two of the ETC complexes are believed to contribute most to the production of ROS: Complex I and complex III [25,26,27,28,29]. Both complexes directly interact with ubiquinone (coenzyme Q_10_) or its reduced form ubiquinol by mediating the conversion to ubiquinol by transferring electrons to it (complex I) or reverting this (complex III), with semiquinone as an intermediate (Figure 3). A functional defect in either complex I or III allows for ubiquinol to be oxidized in an uncontrolled manner by molecular oxygen, enabling the production of superoxide radicals (O_2_^–^), that generate further reactive oxygen species such as hydroxyl radicals and hydrogen peroxide (H_2_O_2_). In humans, the mtDNA encodes seven (ND1, ND2, ND3, ND4, ND4L, ND5, ND6) out of the 45 subunits that compose complex I, and all of these mitochondrially encoded subunits have been implicated in increased ROS production. 

Flavin mononucleotide (FMN) in complex I oxidizes the redox equivalent NADH and transfers the electrons into a series of iron-sulphur clusters that pass electrons into the membrane domain of complex I, where they are eventually transferred to a mobile molecule of ubiquinone, reducing it to ubiquinol. Ubiquinol travels within the inner mitochondrial membrane to complex III. There, two molecules of ubiquinol are processed successively to reduce two molecules of cytochrome c and one bound molecule of ubiquinone. The conversion of ubiquinone to ubiquinol and vice versa progresses through the radical intermediate semiquinone. Presumably, superoxide radicals are produced mainly when electrons leak from semiquinone or reduced flavin onto molecular oxygen.

**Figure 4:**
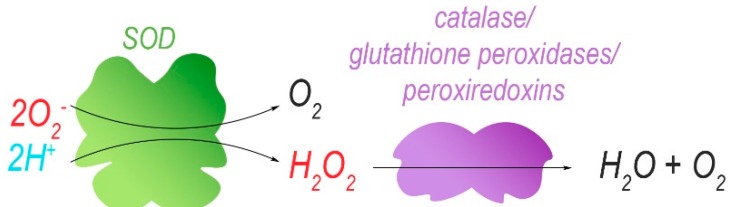
Diagram of breakdown of superoxide into H_2_O_2_ and the enzymes that catalyse each step.

**Figure 5:**
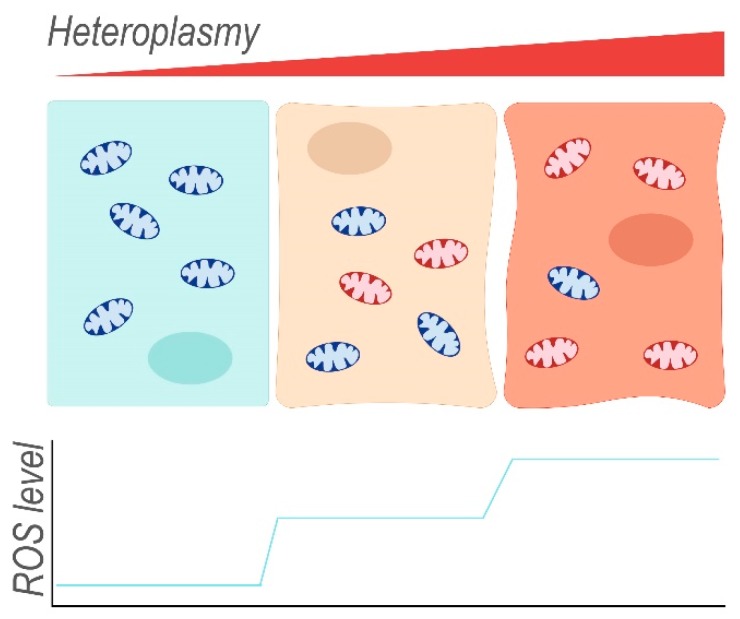
Concept of heteroplasmy and increasing levels of reactive oxygen species (ROS) associated with increasing heteroplasmy.

Isolated mitochondria harbouring mutations in ND5 (m.12417insA) have been re-introduced into cells that were depleted of their own mtDNA to obtain cellular hybrids (cybrids). These cells have enhanced levels (increase of 20%) of mitochondrial superoxide, as determined by using the MitoSox red dye [30]. The superoxide radical is eventually converted to H_2_O and O_2_, with H_2_O_2_ as a ROS-intermediate of this reaction. However, dichlorofluorescein acetate (DCFDA) fluorescence indicated no difference in cellular H_2_O_2_ levels between control and cybrids unless the ND5 mutation reached homoplasmy. DCFDA fluorescence is dependent on the presence of ROS in the cytoplasm, the most abundant of which is H_2_O_2_. Therefore, and because low concentrations of H_2_O_2_ display a linear correlation with DCFDA fluorescence, DCFDA fluorescence intensity is often understood as an equivalent of H_2_O_2_ concentration. At homoplasmy, the ND5 mutation may enhance the cellular concentrations of H_2_O_2_, possibly via increased production of superoxide that exceeds the processing capacity of ROS-protective mechanisms. Indeed, the superoxide dismutases SOD1 and SOD2 that catalyse the conversion of O_2_^–^ into H_2_O_2_, as well as other antioxidants (catalase, glutathione peroxidase Gpx4) (Figure 4), were upregulated in both cybrid lines compared to control, presumably in response to increased ROS production. In mouse cybrids, another ND5 mutation (m.C12081A) that induces a premature stop codon (Arg116Stop) results in a positive correlation between the level of heteroplasmy (16–100%) and their sensitivity towards 5-tert-butyl-hydrogen peroxide, a mimetic of H_2_O_2_ [30]. This suggests that the cybrids with the highest heteroplasmy levels produce the most ROS (Figure 5) and that endogenous protective mechanisms are already at full capacity and less able to deal with exogenous oxidative insults. Indeed, at heteroplasmy levels of 88% and 100%, cybrid viability decreased considerably in the presence of 5-tert-butyl-hydrogen peroxide when compared to lower heteroplasmy levels where ~80% of the cybrid cells were resistant to treatment.

Superoxide ion radicals and protons are converted to hydrogen peroxide by superoxide dismutases (SODs). The reactive hydrogen peroxide is further catalytically degraded to water and molecular oxygen.

Mitochondria containing mainly wild-type mtDNA (blue) occur together with mitochondria harbouring damaged mtDNA (red) within the same cell. The ratio of mutant to wild-type mtDNA (heteroplasmy) can differ between individual mitochondria of a single cell and also between different cells. With increasing heteroplasmy, the fraction of dysfunctional mitochondria that leak ROS increases, thereby enhancing endogenous oxidative stress of the cell.

Mutations in mtDNA-encoded complex I components have been strongly linked with the growth of various cancers through enhanced ROS production. For example, in transformed OKF6 keratinocytes, an ND2 transgene harbouring a mutation (m.G4776A, Ala153Thr) previously associated with head and neck squamous cell carcinomas caused a 1.2-fold increase in the fluorescence of DCFDA, compared to controls [31]. Further experiments with transformed head and neck squamous cell carcinoma and HeLa cervical cancer cells suggested that this mtDNA mutation promoted cancer growth through increased ROS production [31].

A co-occurrence of multiple mtDNA mutations affecting several complexes complicates the identification of ROS-causing or pathogenic mutations. In thyroid oncocytic XTC.UC1 cells, an almost 4-fold increase in DCFDA fluorescence compared to non-oncocytic TPC-1 cells was observed, indicating elevated levels of ROS [32]. This was attributed to the compounded effects of an insertion that induces a premature stop codon in the ND4 gene (m.3571insC, frameshift Gly101X), and a missense mutation in CytB (m.G15557A, Glu271Lys, complex III) that occurred along with a number of silent mutations [32]. Neither of these mutations had been described in healthy subjects before, and while both of them affect complexes that are proposed to be the primary sources of mitochondrial ROS, it remains difficult to determine which of the mutations is causative for the increased peroxide production, or whether both contribute. Either mutation had a high level of heteroplasmy (94.3% for ND4, 70.3% for CytB). 

Further evidence of mtDNA mutations being associated with malignant neoplasms and driving the progression of cancer concerns the ND6 gene, which encodes the core subunit 6 of complex I. The m.G13997A (Pro25Leu) mutation of the ND6 subunit is found in particularly aggressive murine Lewis lung carcinoma cell lines with a high metastatic potential [33,34]. Less aggressive cell lines of this cancer did not carry this mutation. Notably, the mutated residue is highly conserved between human, mouse, fish, frog, chicken, cow and fly, indicating its crucial importance. An increased fraction of the cells of the aggressive cell lines displayed a high H_2_O_2_ production, indicated by DCFDA labelling [33]. Furthermore, completely exchanging the population of mitochondria from less aggressive murine Lewis lung carcinoma cell lines with mitochondria from aggressive lines resulted in a concomitant increase in H_2_O_2_ production. The opposite was true when mitochondria from the less aggressive cells were swapped into aggressive cells, suggesting a direct connection between the m.G13997A mtDNA mutation, H_2_O_2_ over-production, and the aggressiveness of the cancer. In addition, investigation of two fibrosarcoma cell lines confirmed that a frameshift mutation in the ND6 gene (m.13885insC, frameshift) correlated with high ROS production and a high metastatic potential [34]. Subsequently, Yuan et al. surveyed 26 ND6 mutations found in human lung adenocarcinoma and identified a correlation of missense and nonsense mutations with cancer grade, stage and metastatic status [35]. Missense mutations were found to reduce complex I activity and increase ROS production; both effects were found to be more severe with nonsense mutations.

The apparent importance of ND6 and other mtDNA-encoded complex I subunits in disease is not restricted to cancer. Neurodegenerative diseases such as Leber’s hereditary optic neuropathy (LHON) have also been linked to ND6, ND1 and ND4 defects that have been determined to enhance ROS production. With the aim to characterise a mutation detected in human LHON patients, ND6 m.G13997A (Pro25Leu) mice were generated by fusing enucleated cells carrying the mtDNA mutation to mouse embryonic stem cells previously depleted of their original mtDNA [36]. The resulting strain suffers from damage to the retinal ganglion cells, which have abnormal mitochondrial morphology and altered complex I activity. While there was no difference in H_2_O_2_ production between isolated liver mitochondria of mutant and wild-type mice (as measured by the Amplex Red dye), H_2_O_2_ production from submitochondrial particles that lack much of the H_2_O_2_ scavenging system, was significantly elevated in ND6 mutant mice. This was exacerbated in mitochondria isolated from cerebral tissue, where also 3-nitrotyrosine and glial fibrillary protein were increased 2.4-fold and 1.6-fold respectively, indicating oxidative stress and a cellular response to cerebral damage, potentially incurred by ROS. The m.T14484C (Met64Val) mutation in ND6, as well as the m.G3460A mutation in ND1 (Ala52Thr) and m.G11778A mutation in ND4 (Arg340His) have also been associated with increases in ROS production and LHON. Corresponding cybrid lines harbouring each of these mtDNA mutations displayed enhanced mitochondrial superoxide levels as determined by MitoSox staining [37]. Alternative techniques of measuring ROS levels (fluorescence of dihydrorhodamine and Amplex Red) also found similar results for all three mutations implicated in LHON [38,39]. An association of the two LHON mutations in ND1 and ND4 with high levels of ROS has been confirmed in NT2 cells—curiously only after differentiation towards a neuronal fate [40].

Mutations in the ND6 gene can elicit even more devastating and lethal neurodegenerative conditions. Necrotic degeneration of the brain occurs in the context of Leigh syndrome as well as in Leigh-like and bilateral striatal necrosis syndrome, which display high levels of genetic heterogeneity. Gonzalo et al. investigated a m.T14487C mutation in ND6 (Met63Val) that had previously been identified in patients with Leigh-like or bilateral striatal necrosis syndrome and created cybrids from patient thrombocytes by fusing them with the mitochondria-free 143B ρ^0^ cell line [41]. Rates of H_2_O_2_ production in those cybrids were rapidly increased, within 40 min of fusion, while antioxidant enzymes maintained the same level of activity. Malondialdehyde—thiobarbituric acid (MDA-TBA), a measure for lipid peroxidation, was detected at levels of more than double that of control cell lines. Protein carbonylation was unaltered in the cybrids, but DNA damage was assessed using quantitative polymerase chain reaction (qPCR), revealing that in the mutant cybrids, amplification occurs only at 74.4% efficiency compared to control samples, indicating that the template DNA would contain enough damaged sites to reduce the rate of amplification considerably [41].

In addition to pathogenic mutations that are relatively rare in a population, prevalent polymorphisms in mtDNA-encoded complex I subunits have been widely implicated in enhancing susceptibility to or protection from a range of diseases through altered ROS production. Mitochondrial haplogroups are each set apart from the others by a number of characteristic polymorphisms and are often associated to certain geographical locations. For example, the N haplogroup is the (overwhelmingly) dominant haplogroup on the Australian continent and restricted to Australia and East and Southeast Asia. One of the defining polymorphisms of this haplogroup, which encodes a variant of the ND3 gene (m.10398A, ND3:114Thr), increases in ROS production by 52–55% when transfected into HeLa, MDA MB-231, and MCF-7 cells [42]. The most common mtDNA haplogroup in Asia is haplogroup M, which is closely related to haplogroup N, but encodes ND3:114Ala (m.10398G). Transfection with a vector coding the haplogroup M variant increased ROS production only by 14–26%, suggesting that while the procedure by itself might increase ROS production in HeLa cells, the M haplogroup variant induces this effect much less than the N haplogroup variant [42].

In transgenic B6Ntac mice, a polymorphism in the ND4 gene (m.11516G, 450Ser; C57BL/6J-mtAKR reference strain: m.11516A, 450Asn) caused an increased number of bone marrow cells producing mitochondrial superoxide, as well as an approximate two-fold increase in levels of cellular ROS, as indicated by fluorescent peroxide/superoxide dyes [43]. In humans, the neighbouring ND4L gene occurs in variants that promote ROS production under hypoxic conditions and conveys susceptibility to high altitude polycythemia [44]. Cell constructs with the m.10609T variant coding for methionine (ND4L:47Met) produced approximately 1.5-fold more H_2_O_2_ (DCFDA fluorescence) at 3% oxygen levels than constructs with an m.10609C variant coding for threonine in the same position [44]. Under normoxic conditions, no significant difference in ROS production could be observed [44]. The m.10609C polymorphism was present in 12.4% of the sampled population of Han Chinese, it might be associated with longevity in humans [45] and is a defining mutation for mitochondrial haplogroup F1 [46].

## 3. Mitochondrial-Encoded Complex III (Coenzyme Q-Cytochrome C Reductase) Mutations

Complex III is assembled from 11 different subunits, ten of which are nuclear-encoded. The mtDNA encodes cytochrome b (CytB), an integral membrane protein that may play a crucial role in complex III assembly. Mutations in CytB have been found to enhance ROS production in certain cellular contexts. 

A 4 nucleotide deletion in CytB (m.14787–14791del) was detected in a patient suffering from epileptic seizures and parkinsonism. Enucleated fibroblasts of the patient were fused with the mtDNA-less 143B/206 cell line to create cybrids carrying the mutation. These cybrids produce increased amounts of H_2_O_2_, as determined by DCFDA fluorescence [47]. This particular mutation, which occurs at >60% heteroplasmy in the cybrids, presumably interferes with complex III assembly, as Western blotting of mitochondrial protein detects other complex III components at lower than normal levels. Disruption of complex III assembly would potentially interrupt the flow of electrons from ubiquinol, resulting in the inappropriate reduction of molecular oxygen to superoxide and its subsequent degradation to H_2_O_2_. A larger deletion in CytB (m.15642–15662del), which had previously been identified in bladder cancer [48], has also been shown to enhance H_2_O_2_ levels in MB49 bladder cancer cells [49].

In addition, a missense mutation in CytB (m.T14849C, Ser35Pro), which was detected in a patient presenting with isolated complex III deficiency along with two known, homoplasmic polymorphisms (m.C14770T, synonymous and m.A15326G, Thr194Ala), caused symptoms of increased ROS levels in the patient [50]. This included elevated levels of urinary leukotriene E_4_, indicating increased lipid peroxidation in vivo, a slightly reduced total radical-trapping antioxidant parameter of plasma (TRAP) as well as decreased concentrations of α-tocopherol in muscle, indicating a reduced capacity to negotiate higher levels of ROS. The patient suffered from septo-optic dysplasia, hypertrophic cardiomyopathy, rhabdomyolysis and exercise intolerance as well as developmental delays. While this is a drastic phenotype attributable to a CytB mutation, not all changes in this gene necessarily result in a severe increase in ROS production. For example, a CytB mutant mouse strain (m.A15124G, Ile27Val) displayed no significant difference in mitochondrial superoxide and cellular superoxide production, with unimpaired ATP levels [43].

## 4. Mitochondrial-Encoded Complex IV (Cytochrome C Oxidase) Mutations

Complex IV consists of 14 protein subunits, three of which are mitochondrially encoded (CO1, CO2, and CO3). Similarly to complex I and complex III mutations, mutations of complex IV subunits are associated with cancer and elevated ROS levels. The m.T6124C (CO1:Met74Thr) transition was detected in a prostate cancer patient, and caused a marked increase in DCFDA fluorescence, both in a patient-derived lymphoblast cell line compared to control patients, as well as in cybrid lines with the same nuclear genome as wild-type cybrids [51].

In addition, while investigating the inheritance of mtDNA mutations that confer ETC defects of different degrees, a missense mutation in the CO1 gene (m.T6589C, Val421Ala) was assessed in different cell and cybrid lines of murine L cells. Cell lines homoplasmic for the CO1 mutation displayed 3-fold increased DCFDA fluorescence intensities compared to cells that harbour wild-type mtDNA [52]. Based on these cell/cybrid lines, a mouse model was established that was homoplasmic for the CO1 mutation, which consistently caused myopathy and cardiomyopathy [52]. A secondary mutation in ND6 was also present in these mice, but did not appear to contribute to pathogenic symptoms, as only low heteroplasmy levels (14–16%) could be established [52]. 

As CO1, CO2 and CO3 are core proteins of complex IV, mutations in any of them can impair the correct assembly of complex IV and may enhance the production of ROS. The m.G6930A substitution that introduces a premature stop codon in CO1 (Gly343Stop), leads to the loss of the last 170 amino acids of the 511 amino acids long protein [53] and prevents the assembly of complex IV [54]. The patient presenting with this mutation suffered from deafness and optic and muscle atrophy, causing weakness, ataxia and visual defects. No significant difference in ROS levels (DCFDA fluorescence), the expression of antioxidant enzymes (Western blotting), or lipids oxidative damage (MDA-TBA assay) was found in 143B ρ^0^ cybrids with the mutation compared to wild-type mtDNA cybrids [55], however, an increased susceptibility towards H_2_O_2_ treatment was observed [56]. 

Similar perturbations in complex IV assembly seem to be caused by pathogenic mutations in CO2. The nonsense m.G7896A CO2 mutation (Trp104Stop), for example, was initially detected in a 3-year old patient with an early-onset complex IV deficiency. Symptoms including psychomotor delay, failure to thrive and cardiac hypertrophy presented from an age of 3 months, later developing into comprised cerebral atrophy and pigmentary retinopathy [57]. Cybrid cell lines homoplasmic for the mutation were unable to assemble complex IV or any CO-containing supercomplex [58]. Similarly, the m.T7671A mutation in CO2 (Met29Lys), which was identified in a patient presenting with myopathy and lactic acidosis [59], was shown to inhibit correct complex IV assembly. Perturbations to complex IV assembly has recently been demonstrated to enhance mitochondrial superoxide levels (as measure with MitoSox), decrease ATP production and increase rates of senescence and aptoptosis [60], suggesting that the above pathogenic mutations may do the same. Finally, mutations in CO3 that are associated with mitochondrial encephalomyopathy, lactic acidosis, and stroke-like episodes (MELAS) cause increases in ROS production. The m.T9957C mutation (Phe251Leu) induces a 1.5-fold increase in DCFDA fluorescence in cybrids compared to the 143B parent cell line that retained its original mitochondria [61]. 

## 5. Mitochondrial-Encoded Complex V (ATP Synthase) Mutations

The last step in the OXPHOS chain is mediated by complex V, an ensemble that consists of a central rotor complex that synthesizes ATP in a proton electrochemical gradient driven reaction. The mtDNA encodes 2 subunits of complex V, ATP6 and ATP8. The m.T8993G mutation in the ATP6 gene (Leu156Arg) is associated with neurogenic ataxia retinitis pigmentosa (NARP) as well as maternally inherited Leigh’s syndrome (MILS) [62]. Cybrids from 143B ρ^0^ cells and blood platelets produce increased amounts of H_2_O_2_ in proportion to the level of heteroplasmy of the mutation (~2 fold for 50% heteroplasmy, and ~4 fold at 100%) [63]. Superoxide production was also increased in a m.T8993G prostate cancer mouse model [64]. Lipid hyperoxidation, measured by malondialdehyde and 4-hydroxyalkenals levels, was also increased markedly in the 143B cybrid cells and the activity of the mitochondrial superoxide dismutase, MnSOD, was increased 1.7-fold for cybrids harbouring the m.T8993G mutation at 50% heteroplasmy, and almost 3.5-fold for homoplasmic cybrid cells [65]. A similar finding has been made in NARP patient fibroblasts carrying the m.T8993G mutation, where both MnSOD and the cytoplasmic CuZnSOD activity were three times higher than in control samples [66]. This suggests that mtDNA-encoded defects in complex V increase ROS production, which has been proposed to play a major role in the pathogenesis of NARP/MILS. Indeed, the antioxidants N-acetylcysteine and dihydrolipoic acid have been tested in fibroblasts harbouring 97% m.T8993G mutant mtDNA and have been shown to significantly improve mitochondrial respiration and ATP synthesis in these cells [65]. The substitution of T8993 by cytosine instead of guanosine (m.T8993C, Leu156Pro) results in further pronounced increase in ROS production compared to the m.T8993G mutation in human patient lymphocytes [67] and causes a typical phenotype of episodic weakness and motor neuropathy [68].

In a set of patients that shared clinical periodic paralyses combined with other individual symptoms, other ATP6 mutations (m.T9185C, Leu220Pro and m.T9176C, Leu217Pro) were detected that were associated with increased oxidative stress [68]. Interestingly, this panel of individuals also included a patient with a mutation of the ATP8 gene (m.T8403C, Ile13Thr), suggesting that this subunit might also contribute to mitochondrial ROS production. In mice, a polymorphism of ATP8 (m.G7778T, Asp5Tyr) causes ATP levels 3-fold lower than in control animals and an increase in H_2_O_2_ production, determined by Amplex Red dye in isolated mitochondria of the spleen [69]. The affected mitochondria appear swollen [70] and a significant increase of MitoSox fluorescence could be detected in pancreatic islets of affected mice compared to control animals [69]. However, common polymorphisms in ATP8 (m.C8414T, Leu17Phe) in Han Chinese populations do not induce altered ROS production at normoxic or hypoxic conditions [44]. 

## 6. Mitochondrial-Encoded tRNA and rRNA Mutations

As mentioned above, the aberrant assembly of some respiratory complexes can lead to enhanced ROS production. It is thus unsurprising that mutations in mtDNA-encoded tRNAs and rRNAs, which are required for the translation of the mtDNA-encoded ETC subunits, have been heavily implicated in the production of ROS, as well as disease. This is supported by the findings of Cruz-Bermudes et al. (2015), which demonstrated that 143B ρ^0^ cybrid lines harbouring a mutation in the tRNA for lysine (tRNA^lys^: m.G8363A) displayed enhanced mitochondrial superoxide levels and slightly elevated cytoplasmic ROS levels, as determined by MitoSox and DCFDA fluorescence respectively [37]. The m.A8344G mutation, which also perturbs tRNA^lys^ and was obtained from a myoclonic epilepsy with ragged red fibres (MERRF) patient, causes a 68% increase in DCFDA fluorescence in cybrids [55]. Similar results were also obtained by studying skin fibroblasts from other patients with the same mutation [71]. In the same study, a mutation in the tRNA^leu^ gene (m.A3243G), which was isolated from a patient with MELAS, resulted in an 83% increase in DCFDA fluorescence, indicating markedly enhanced H_2_O_2_ production [71]. Similarly, other mutations in tRNA^leu^ (m.T3253C and m.A12308G) have been shown in cybrids to enhance ROS production [72,73]. Interestingly, the m.A12308G mutation is localised to the second tRNA^leu^ locus within the mtDNA and was isolated from the MDA-MB-435 breast cancer cell line [73]. This suggests that the mutations in either of the two genes coding for tRNA^leu^ may act dominantly to disrupt the translation of mtDNA-encoded proteins, resulting in ROS production and disease.

Perhaps exemplifying the importance of tRNA integrity in the production of ROS, a mouse model harbouring multiple mtDNA mutations in both tRNAs and protein-coding genes was found to overproduce ROS in a manner solely dependent upon the tRNA mutation. The comparison of several mouse strains that differ in the length of a poly-A-stretch insertion within the tRNA^arg^ gene (m.9821insA 8A/9A/10A) revealed that cybrids with a 10-A-stretch exhibited significantly increased H_2_O_2_ production (DCFDA fluorescence) compared to 8-A- and 9-A-stretch cybrids [74]. The 10A and 9A variants of this stretch are often coincident with polymorphisms in the ND3 and CO3 genes (compared to C57BL/6J mice: m.T9461C, synonymous, and m.G9348A (CO3:V248I)) [43,75]. However, only in the presence of the 10A-stretch are increases in ROS production observed [74], indicating that the major contributor to oxidative stress is the level of perturbation to tRNA^arg^. Interestingly, one of the studies characterising mice of the 10A haplogroup reports differences in ROS levels only beyond nine months of age [75]. This coincided with increased liver tissue degeneration, inflammation and fibrosis and altered mitochondrial morphology, suggesting that the pathogenic potential of some tRNA mtDNA mutations may become apparent only with age [75].

An increase in ROS production has been reported also for mutations in tRNA^gly^ (m.T10003C) [72,76], tRNA^met^ (m.C4467A) [77], tRNA^his^ (T12201C) [78], tRNA^trp^ (m.C5541T) [79], tRNA^ala^ (m.T5655C), tRNA^asp^ (m.A7551G), tRNA^glu^ (m.A14692G), and slightly for tRNA^thr^ (m.A15909G), which are often accompanied by a corresponding decrease in ATP production [72]. In addition, a patient bearing a tRNA^val^ mutation (m.A1630G) suffering from gastrointestinal dysmotility and cachexia, presented with increased serum ferritin and lowered transferrin levels [80], both previously associated with a high ROS burden [81,82]. Moreover, a deficiency in negotiating exogenous oxidative stress was found in lymphoblastoid cell lines harbouring a tRNA^ile^ mutation (m.A4263G) [83], as well as in cybrids carrying either tRNA^thr^ (m.A15909G) [84] or tRNA^met^ (m.A4435G) mutations [85]. This implies that cellular ROS-protective mechanisms have a reduced capacity to ameliorate the additional external stress due to the presumably already elevated endogenous ROS levels.

Similarly to tRNAs, defects in the two mtDNA-encoded rRNAs, 16S rRNA and 12S rRNA, affect the synthesis of mitochondrially encoded proteins and therefore the assembly of OXPHOS machinery. Mutations of the 12S rRNA, which is a component of the small subunit of the mitochondrial ribosome, affect RNA secondary structure and have been widely associated with hearing loss and ototoxicity of certain antibiotics [86,87]. In an attempt to establish a mechanistic link between one of the most common 12S rRNA mutations in hearing loss and a greater susceptibility to deafness, cybrids of three hearing loss patients with a m.C1494T mutation were compared with cybrids harbouring the mitochondria of control subjects [88]. Western blotting revealed consistently decreased levels of mitochondrially encoded proteins in the mutant cybrid lines, reduced ATP production and an increased number of MitoSox positive cells, indicating enhanced levels of mitochondrial superoxide [88]. The m.A1555G mutation in 12S rRNA is also associated with maternally inherited deafness and doubled mitochondrial superoxide production in cybrids compared to wild-type controls; and also induces caspase 3/7 activity, indicating the initiation of apoptosis [89]. 

A mutation in 16S rRNA (m.A2905G), which forms part of the large subunit of the mitochondrial ribosome, was detected along with three other mutations in the ATP6 and ND4 genes in a pancreas cancer cell line; however, while the mtDNA mutations confer chemoresistance to common cancer drugs, Satoshi et al. (2009) indicate that they did not observe any increase in ROS production of mutant cybrids compared to wild-type controls [90]. In contrast, a significant, more than 1.5-fold increase in DCFDA fluorescence was observed in lymphoblastoid cell lines established from hypertrophic cardiomyopathy patients with a m.T2336C substitution in the 16S rRNA gene compared both to a 143B cell control and corresponding cell lines obtained from control subjects of the same haplogroup [91]. The oxygen consumption rate and mitochondrial ATP synthesis were reduced as well, indicating mitochondrial dysfunction [91].

## 7. Mutations in Polymerase γ (Nuclear-Encoded)

Above, we have described the impact of particular individual mtDNA lesions upon ROS production. However, the accumulation of multiple, distinct mtDNA mutations across multiple genomes has been predicted to occur during the ageing process in cells [6,7,92,93,94,95,96]. The myriad of distinct and perhaps extremely rare mtDNA lesions is difficult to detect in an individual with the use of current technology. However, given what we know about individual mutations, it is reasonable to predict that the compounded effect of acquired mtDNA mutations over time would result in a progressive increase in ROS production and a decrease in ATP synthesis. ROS-induced increases in damage to proteins, lipids and nucleic acids and a loss of cellular functions due to a lack of ATP may eventually lead to cellular dysfunction, senescence and apoptosis. Successively, tissue functionality would be compromised, causing the symptoms of ageing in the individual. However, this mitochondrial theory of ageing has suffered from a lack of direct experimental evidence.

Replication errors that occur during mtDNA duplication are a likely cause for most acquired mtDNA mutations. Replication of the mitochondrial genome is performed exclusively by DNA polymerase γ, a nuclear-encoded enzyme with a catalytic subunit (POLGA) that performs the polymerase and exonuclease activities, and an accessory subunit (POLG2) that enhances processivity. Accelerating the accumulation of de novo mtDNA mutations has been achieved in mice by mutating the POLGA subunit of DNA polymerase γ, affecting the exonuclease activity and thus the proofreading capacity of the polymerase [97]. Compared to wild-type littermates, POLGA mutant mice display a 3- to 8-fold increase in the frequency of somatic mtDNA mutations and display premature signs of ageing as well as a shortened lifespan [97]. However, while there was evidence of nuclear DNA damage (TUNEL stain indicating double strand breaks) and cell death (presence of cleaved caspase 3), the POLGA mutant mice did not display increased ROS production in heart and liver tissue, or any signs of increased oxidative stress. Similar results were obtained by Trifunovic et al., which reported an increase in mtDNA mutation load in POLGA mice but only a small increase in carbonylated mitochondrial proteins, suggesting that there were no drastic differences in mitochondrial ROS production [98]. In addition to the observations made at 40 weeks of age, mouse embryonal fibroblasts obtained from POLGA mice do not seem to produce more H_2_O_2_, nor are they more vulnerable towards H_2_O_2_ treatment than wild-type embryonal fibroblasts. 

Logan et al. propose that mitochondrial ROS production is tightly regulated within cells, and ex vivo experiments may not provide the appropriate conditions to accurately reflect mitochondrial ROS in vivo [99]. To overcome this problem, the MitoB probe can be injected in live animals where it accumulates in mitochondria. Its reaction with H_2_O_2_ to MitoP was assessed by mass spectrometry, indicating higher ROS levels in mature POLGA mutator mice than in wild-type mice [99]. The challenge of detecting differences in ROS levels between POLGA and wild-type mice is further compounded by the circumstance that the activity of the exonuclease domain is required to create ligatable mtDNA ends after replication to achieve its closed circular confirmation [100]. Linearization of the mtDNA results in its degradation, so a failure to ligate due to a deficiency of the exonuclease activity might affect transcription from the unligated mtDNA. While the mutated DNA is detectable in sequencing, transcription of the compromised mtDNA may be inefficient. As such, mutant subunits may be produced and incorporated into the OXPHOS machinery not very frequently, which may reduce and delay aberrant ROS production.

A different polymerase γ mutation in the catalytic subunit POLGA (Tyr955Cys) that in humans, causes Chronic Progressive External Ophthalmoplegia (CPEO), does not impact the exonuclease function, but instead affects the polymerase activity. Placed close to the catalytic site, this residue presumably is involved in the binding of nucleoside triphosphates. The Tyr955Cys mutation reduces the processivity of polymerase γ and in absence of the proofreading exonuclease activity, increases the frequency of misinsertions during replication [101]. The mouse lines established by Lewis et al. express POLGA^Tyr955Cys^ under a tissue-specific promoter restricted to the cardiac muscle. While the mice display no obvious phenotypic variations, their life expectancy was reduced drastically and the mice suffered from cardiac enlargement and cardiomyopathy [102]. Alterations in mitochondrial morphology, such as mitochondrial swelling, dissolution of the christae and altered structure of the matrix were reported. In contrast to the mutations of the exonuclease activity, the PolgA^Tyr955Cys^ mutation does increase the frequency of the oxidative DNA damage marker 8-OHdG by approximately threefold, suggesting that ROS production is elevated when the rate of mtDNA mutations is enhanced.

## 8. Conclusions

While the biological importance of ROS in the biogenesis of mtDNA mutations is far from clear, the above experimental evidence indicates that diverse mutations in almost all mtDNA-encoded genes can enhance ROS production in a number of cellular settings. ROS have important physiological roles in signalling and immunity, however the pathological consequences of excessive oxidative stress originating from mtDNA mutations are wide, complex, and varied. Although the involvement of oxidative stress in ageing itself is controversial, in many instances, disorders thought to be caused by excessive oxidative stress generated by the mitochondria are associated with ageing-related pathologies, such as neurodegeneration [103]. Mutations in the mitochondrial genome have been found to accumulate in somatic tissues during normal ageing [104], as has an increase in ROS production and biomarkers of its damage [105], however, demonstrating causality between the two has been challenging as has their direct links with ageing and longevity. The mtDNA mutational landscape is open to spatiotemporal fluctuations during a lifetime [4], and it is likely that all individuals harbour mtDNA mutations, even if at undetectable levels. As a consequence, mitochondrial ROS production may be highly dynamic and highly variable between cells. A future research goal will be to unravel the intricacies of these relationships.
